# Cryoprotectant treatment tests on three morphologically diverse marine dinoflagellates and the cryopreservation of *Breviolum* sp. (Symbiodiniaceae)

**DOI:** 10.1038/s41598-021-04227-2

**Published:** 2022-01-13

**Authors:** Joseph Kanyi Kihika, Susanna A. Wood, Lesley Rhodes, Kirsty F. Smith, Lucy Thompson, Sarah Challenger, Ken G. Ryan

**Affiliations:** 1 Cawthron Institute, Private Bag 2, Nelson, 7042 New Zealand; 2 School of Biological Sciences, Victoria University of Wellington, PO Box 600, Wellington, 6140 New Zealand; 3 School of Biological Sciences, University of Auckland, Private Bag 92019, Auckland, 1142 New Zealand

**Keywords:** Biological techniques, Cell biology

## Abstract

Dinoflagellates are among the most diverse group of microalgae. Many dinoflagellate species have been isolated and cultured, and these are used for scientific, industrial, pharmaceutical, and agricultural applications. Maintaining cultures is time-consuming, expensive, and there is a risk of contamination or genetic drift. Cryopreservation offers an efficient means for their long-term preservation. Cryopreservation of larger dinoflagellate species is challenging and to date there has been only limited success. In this study, we explored the effect of cryoprotectant agents (CPAs) and freezing methods on three species: *Vulcanodinium rugosum*, *Alexandrium pacificum* and *Breviolum* sp. A total of 12 CPAs were assessed at concentrations between 5 and 15%, as well as in combination with dimethyl sulfoxide (DMSO) and other non-penetrating CPAs. Two freezing techniques were employed: rapid freezing and controlled-rate freezing. *Breviolum* sp. was successfully cryopreserved using 15% DMSO. Despite exploring different CPAs and optimizing the freezing techniques, we were unable to successfully cryopreserve *V. rugosum* and *A. pacificum*. For *Breviolum* sp. there was higher cell viability (45.4 ± 2.2%) when using the controlled-rate freezing compared to the rapid freezing technique (10.0 ± 2.8%). This optimized cryopreservation protocol will be of benefit for the cryopreservation of other species from the family Symbiodiniaceae.

## Introduction

Dinoflagellates are eukaryotic microorganisms that are ecologically diverse and inhabit a wide range of aquatic ecosystems. They occupy planktonic, meroplanktonic, and benthic habitats, and are important contributors to primary production^1^. Some dinoflagellate species form Harmful Algal Blooms (HABs) commonly known as “red tides” which can negatively affect marine life and human health^2,3^. HABs cause direct and indirect economic losses through the costs of preventative measures, monitoring efforts and revenue losses due to the reduction in tourism and recreational activities^4^.

Cultures of dinoflagellates are used in research programmes worldwide including in the development of certified reference standards for phytoplankton monitoring, development and testing of molecular DNA assays, and the production of a diverse array of bioactive secondary compounds that include toxins with pharmaceutical potential^5^. Some species, such as those from the family Symbiodiniaceae, are currently being used globally to understand a multitude of stressors, including ocean warming, ocean acidification, and eutrophication, all of which have caused a decline in coral health and cover in recent decades^6^. Three dinoflagellates species that are of high interest worldwide for the above reasons are *Vulcanodinium rugosum*^7^, *Alexandrium pacificum*^8^ and *Breviolum* sp.^9^.

*Vulcanodinium rugosum* is a peridinoid dinoflagellate first identified in the non-motile life stage from northern New Zealand in 2008^10^ and formally described from the French Mediterranean coast^7^. It is characterized by a distinct apical pore complex on the motile cell and a life cycle dominated by a non-motile stage of spherical cells that are loosely associated with benthic sediments. This dinoflagellate produces the neurotoxins pinnatoxin E, F and G, which accumulate in shellfish in Japan, Australia, New Zealand, China, and France^11–13^. It also produces the polycyclic ether portimine, a highly bioactive secondary metabolite^14^.

*Alexandrium* is a wide-spread, cosmopolitan dinoflagellate genus and many species are known to form extensive harmful blooms^15^. *Alexandrium pacificum* is endemic to the East Asian temperate regions^16–18^, New Zealand^19^, Tasmania and New South Wales in Australia^19,20^. It produces saxitoxins that accumulate in shellfish and can cause Paralytic Shellfish Poisoning in humans if contaminated shellfish are consumed^19,21^.

Dinoflagellates belonging to the family Symbiodiniaceae are keystone taxa found in coral reef ecosystems. They form crucial endo-symbiotic associations with corals and other invertebrates, such as clams and anemones, as well as with single-celled eukaryotes such as foraminifera and ciliates^22,23^. They are a genetically diverse family of unicellular dinoflagellate symbionts that comprise at least nine divergent clades^6,24^. Some Symbiodiniaceae have been found to dominate coral communities that inhabit shallow waters (1–15 m) across the Greater Caribbean^9,25^, specifically *Breviolum* spp. that are endemic to the Atlantic Ocean^25^. *Breviolum* species form mutualistic interactions with the corals and therefore conserving these dinoflagellates will be a critical factor in coral reef conservation^25,26^.

Dinoflagellate cultures are generally maintained by serial sub-culturing of living cells into fresh liquid media at regular intervals. This is both labour intensive and expensive, and there is a high risk of contamination to healthy dinoflagellate cultures from other microorganisms that can lead to eventual culture loss^5^. To protect and preserve valuable dinoflagellate species, cryopreservation has been touted as the most reliable technique to ensure their long-term genetic stability and to reduce storage and regular maintenance costs^5,27,28^.

Cryopreservation involves the storage of samples at ultralow temperatures (− 196 °C) in liquid nitrogen, and it has been successfully applied in the long-term preservation of valuable microalgal species^5,29,30^. However, attempts to have a well optimized and uniform cryopreservation protocol that can be applied to all microalgae have been unsuccessful^31^. In most cases, success in cryopreservation depends on the application of suitable rates of cooling that minimize the formation of ice crystals during freezing and the use of non-toxic cryoprotectants^32,33^. Cryoprotectant agents (CPAs) such as glycerol, dimethyl sulfoxide (DMSO) and methanol are common cell-penetrating cryoprotectants used in many marine microalgae species^5,34^. DMSO is favoured for the cryopreservation of a range of marine algae species^5,30,34^. The combination of a rapidly penetrating and a non-penetrating CPA for cryopreservation may provide a greater protective effect than when each type of CPA is used individually^35–37^. DMSO is the most effective and rapidly penetrating CPA for marine microalgae compared to glycerol and other CPAs^30,35^ and it has been successfully combined with sorbitol in the cryopreservation of the brown macroalga *Saccharina latissima*^36^.

Cryopreservation of various small microalgae from different classes has been successful, but large unicellular and colony forming marine microalgal species, especially those containing large vacuoles, have been difficult to cryopreserve^5,38^. Similarly, some large toxin-producing dinoflagellates have never been cryopreserved successfully^5^. Previously, four species of Symbiodiniaceae from three different clades have been cryopreserved: a clade D strain using ethylene glycol or propylene glycol^39,40^, a clade B strain using methanol and ethanol^41^, a *Breviolum* sp. using propylene glycol and methanol^26^ and a clade G strain using ethanol combined with sucrose^37^.

The present study describes the treatment of three different dinoflagellates with various types of single and combined CPAs in an attempt to develop an optimized protocol for their cryopreservation. Large toxin-producing dinoflagellates have been difficult to cryopreserve^5^ and there is an urgent need to further study those types of CPAs that might enhance their cryopreservation. Identifying the best type of CPA and its final concentration that is not toxic to these key marine dinoflagellates may enhance their survival during freezing. In this study we tested and identified numerous CPAs but were only able to successfully cryopreserve *Breviolum* sp. Our research on CPAs paves the way for more studies to explore different approaches for cryopreserving large dinoflagellates. Increasing the number of dinoflagellate cultures that can be cryopreserved will enable the safeguarding of these precious resources and allow further research and commercial applications.

## Materials and methods

### Isolates studied and their cultivation conditions

Three dinoflagellate cultures, *V. rugosum* (isolate CAWD296), *A. pacificum* (isolate CAWD234) and *Breviolum* sp. (isolate CAWD197), were obtained from the Cawthron Institute Culture Collection of Microalgae (CICCM; http://cultures.cawthron.org.nz/ciccm/). *Amphidinium carterae* (isolate CAWD57) was used as a positive control during the cryopreservation procedure as this strain has been successfully cryopreserved previously^5^. *Vulcanodinium rugosum* (CAWD296), was grown in nutrient enriched 50% GP medium^42^ and *Breviolum* sp. (CAWD197) in F/2 /L1 growth medium^43,44^. *Alexandrium pacificum* (isolate CAWD234) and *A. carterae* (CAWD57) were grown in nutrient enriched GP medium^45^. All cultures were grown in 12:12 light: dark cycle under 100 µmol m^−2^ s^−1^ photosynthetically active radiation (PAR). *Vulcanodinium rugosum* and *Breviolum* sp. were both grown at 25 °C and *A. pacificum* and *A. carterae* at 18 °C^5,46^. All cultures were harvested during their late exponential phase prior to CPA treatments and freezing experiments.

### Types and combinations of cryoprotectant agents

Eight single CPAs commonly used in cryopreserving different types of microalgae were tested empirically to determine the optimal working concentrations^5,30,35^ for the three dinoflagellate species. Methanol was preferred to ethanol because it is more effective, penetrates the cells more easily and is less toxic^30,35^. The CPAs used were; DMSO (Purity GC ≥ 99.9%, Sigma-Aldrich, Japan), Methanol (MeOH; HPLC grade, ≥ 99.9%, Sigma-Aldrich, Germany), Glycerol (Sigma-Aldrich, Malaysia), Propylene glycol (PG; Sigma-Aldrich, Singapore), Diethylene glycol (DEG; Sigma-Aldrich, USA), Ethylene glycol (EG; spectrophotometric grade, ≥ 99%, Sigma-Aldrich, Malaysia), Polyvinylpyrrolidone (PVP; Sigma-Aldrich, USA) and Polyethylene glycol (PEG; Sigma-Aldrich, Germany). Single CPA solutions were prepared in sterilized growth medium to give final concentrations of 5%, 8%, 10%, 12%, and 15% (v/v or w/v).

For combined CPA treatments, DMSO was preferred because it penetrates the cell membrane and into the intracellular space more easily than glycerol and does not cause excessive bacterial contaminations^30^. DMSO was combined in a ratio of 1:1 with the non-penetrating compounds proline (Sigma-Aldrich, USA), sucrose (BDH, England), sorbitol (Sigma-Aldrich, USA) and glucose (BDH, England). The concentrations for the combined CPAs were prepared for the different species as shown in Table 1.

**Table 1:** Treatment tests using combinations of two cryoprotective agents (CPAs) on the three different dinoflagellate species.

Species	Final working concentrations of CPAs (%)
*Vulcanodinium rugosum* and *Alexandrium pacificum*	5% proline or sucrose or glucose or sorbitol + 5% DMSO
	5% proline or sucrose or glucose or sorbitol + 8% DMSO
	5% proline or sucrose or glucose or sorbitol + 10% DMSO
	8% proline or sucrose or glucose or sorbitol + 5% DMSO
	8% proline or sucrose or glucose or sorbitol + 8% DMSO
	8% proline or sucrose or glucose or sorbitol + 10% DMSO
*Breviolum* sp.	5% proline or sorbitol + 5% DMSO
	5% proline or sorbitol + 8% DMSO
	5% proline or sorbitol + 10% DMSO
	8% proline or sorbitol + 5% DMSO
	8% proline or sorbitol + 8% DMSO
	8% proline or sorbitol + 10% DMSO

### Antibiotic treatments on some cryoprotectant treatment tests

Our study focused on cryoprotectant treatment tests and dinoflagellate cryopreservation. However, we experienced some unwanted bacterial overgrowth in glycerol, PEG, PVP, proline + DMSO, sucrose + DMSO, glucose + DMSO and sorbitol + DMSO treatments, and antibiotics were added to all these treatments. Five antibiotics (Ampicillin, Penicillin-G, Gentamycin, Ciprofloxacin, and Streptomycin) were added separately into 12-well plates with CPA treated non-axenic dinoflagellate cultures (Supplementary Information Table S1). The bacterial growth after CPA treatment was categorized into four different levels depending on the cloudiness of the media (Supplementary Information Table S2). An antibiotics mixture 1 µL of each (Penicillin-G + Gentamycin + Streptomycin) was added to 1 mL of growth media successfully reduced bacterial overgrowth (Supplementary Information Fig. S1) and was used in all further experiments with these dinoflagellate species and the CPAs that encouraged bacterial growth.

### Cryoprotectant agents; treatment and incubation of cultures

Prior to testing each CPA, aliquots (20 mL) of each culture were transferred to a sterile plastic flatbottomed vessel (70 mL Labserv, Thermofisher scientific NZ) in a laminar low cabinet. For *V. rugosum* and *Breviolum* sp. strains, cells attached to the bottom of the culture flask were gently detached using a sterile cell scraper (Falcon, Mexico). An aliquot (1 mL) of each culture was pipetted into 12-well plates (Costar, China). In single CPAs, each CPA was prepared at double the desired final concentration using the species growth medium. For the combined CPAs, each individual CPA concentration was prepared at four times the final concentration. Every minute, 100 µL aliquots of the CPAs were added to the wells with gentle agitation (10 min, RT, 30 µmol m^−2^ s^−1^ PAR) between each addition until a final 1:1 dilution was obtained after the 10th step. The CPA treated cultures were left to equilibrate in complete darkness (30 min, RT). *Alexandrium pacificum* was maintained at 18 °C, and *V. rugosum* and *Breviolum* sp. at 25 °C, under controlled minimal light (56 µmol m^−2^ s^−1^ PAR). The treatments were carried out in triplicate.

### Cryoprotectant agents; impacts on cell morphology and long-term recovery

After incubation for one-week with different CPAs, dinoflagellate cells were categorized as either healthy, unhealthy, or dead and enumerated based on changes in the chlorophyll pigmentation as observed using an inverted microscope (Olympus CK X41 Tokyo, Japan; Supplementary Information Fig. S2). After the one-week incubation, 1 mL of the cells from the CPA treated replicates was transferred into separate sterile plastic flatbottomed vessels with 30 mL of fresh growth media without CPA. The cultures were incubated in their normal growing conditions and the mean girdle diameter of the treated and untreated cells was measured microscopically after one and two-weeks post CPA treatment.

### Freezing protocols

Prior to freezing, aliquots (1 mL) of each dinoflagellate culture were pipetted into 5 mL sterile glass test tubes in a laminar flow cabinet. For each species, 100 µL aliquots of the preferred CPA(s) was added to the tubes with gentle agitation (10 min, RT, 30 µmol m^−2^ s^−1^ PAR) between each addition until a final 1:1 dilution was obtained. After the final addition, the tubes were stoppered, covered with aluminum foil, and left to equilibrate in the dark (30 min). The cultures were aspirated into cryopreservation straws (0.5 cc, IMV, France), plugged with coloured polyvinyl chloride powder, and placed in water (20 °C) to set the powder. The straws were then wiped dry before the freezing procedure. This ramping procedure is the same as used by Rhodes, et al.^5^ for the positive control (*A. carterae*) and was applied to all species in this study.

Two freezing techniques were assessed.

1. Rapid freezing technique: Straws containing the CPA treated dinoflagellate cultures were arranged horizontally on a metal rack fitted onto polystyrene floats. The rack measured (41 × 14 × 4 cm, l × w × h). The rack was place gently over the liquid nitrogen bath (45 × 30 × 6 cm, l × w × h) where it floated for 10 min. The aim of this was to induce rapid freezing of the cells before plunging the straws into liquid nitrogen.
2. Controlled-rate freezing technique: Straws were transferred to a controlled-rate freezer (Cryologic Pty, Mt Waverley, Australia) programmed to cool from 20 to − 40 °C at a rate of 1 °C min^−1^. The straws were held at − 40 °C for 10 min before plunging into liquid nitrogen.

The frozen straws from both experiments were stored in a dewar containing liquid nitrogen for 4 days.

### Thawing procedure

Straws were thawed by plunging into a water bath (20 °C) until all visible ice melted. The straws were then wiped dry with a tissue moistened with 70% ethanol. Working in a laminar flow cabinet, the straw contents were transferred to an empty sterile plastic flatbottomed vessel and individually diluted by stepwise addition of 500 µL of their growth medium each minute for 10 min (total volume 5 mL). The plastic flatbottomed vessels were kept in the dark (30 min) to equilibrate and then a final 5 mL of growth medium was added to provide a 20 × dilution of the initial straw volume. The plastic flatbottomed vessels were kept under dark conditions at 20 °C for 24 to 36 h, followed by a further 48 to 60 h under 27 µmol m^−2^ s^−1^ of red light (OSRAM L18W/60, Germany). Finally, media (40 mL) was added to the plastic flatbottomed vessel and the microalgal cultures were transferred to their normal standard light conditions for growth.

### Assessment of post-thaw cell viability

After thawing, *Breviolum* sp. and *A. carterae* survived and the cells divided into healthy cultures. In *Breviolum* sp., healthy swimming cells were observed five days after thawing and three days in *A. carterae*. *Vulcanodinium rugosum* and *A. pacificum* did not survive after thawing. A viability test was conducted after thawing to determine the survival of *Breviolum* sp. and *A. carterae* cells after freezing. The cells were carefully resuspended in the plastic flatbottomed vessels using a pipette and 1 mL of the cell culture was taken and a serial ten-fold dilution was made using the species growth media for easy determination of the cell concentration. Lugol’s iodine (10 µL) was added and 100 µL of the sample was placed on a glass slide in triplicate and the cells allowed to settle for 30 min. An inverted microscope was used to enumerate viable cells (stained dark brown), and dead cells (colorless).

### Growth curve and viable cell counts

Four straws containing *Breviolum* sp. cells were thawed into four different plastic flatbottomed vessels with equal media volume and growth was monitored. A non-cryopreserved culture of *Breviolum* sp. was used as a control. Before subsampling for cell counts, cultures were resuspended using a pipette. An aliquot was then taken from each plastic flatbottomed vessel, and a serial ten-fold dilution was made using the species growth media for easy determination of the cell concentration. From the serial culture dilutions, 100 µL of the suspensions was dropped on a microscope glass slide and counted in triplicate using the direct-counting method^47^ and an inverted microscope (Olympus CK X41 Tokyo, Japan). Cell counts were undertaken every two days. For the cryopreserved *Breviolum* sp., cell counts were initiated four days after thawing which was when normal cell division was observed. Growth rate (divisions day^−1^) was calculated using the cell density from the exponential portion of the growth curve by least squares regression^48^. Maximum growth rates were calculated for both the cryopreserved and the control cultures, and the highest cell density obtained during the growth period was expressed as the maximum cell density per mL.

### Statistical analysis

All statistical analyses were performed in R (version 4.0.3). For the combined CPA treatments, the mean value of the healthy cells in all the triplicates was calculated and these were visualized in box plots. To determine if there was a significant difference between the diameters of the CPA treated and the untreated dinoflagellate cells, unpaired t-tests were undertaken (p < 0.05) and boxplots used to show the median distribution of the cell diameters. The nonparametric Wilcoxon test was applied to compare maximum growth rate and concentrations between the cryopreserved and control *Breviolum* sp. culture. Error bars in the growth curves were standard error of the mean. Data were expressed as mean ± SD and p-values of less than 0.05 were considered to be statistically significant.

## Results

### Effects of cryoprotectant treatment on cell health—single CPAs

In single CPA treatments, three preferred CPAs were selected for the freezing experiments based on their limited impacts on the cell morphology and cell health in each species. The results from the top three CPAs for each species are described in detail here. The full results of all other cryoprotectant treatments are shown in Supplementary Information Tables S3 to S5.

There was 100% cell survival in *V. rugosum,* after treatments in 5% glycerol, DMSO and DEG (Table S3). When the CPA concentration was increased to 8%, there was a 100% cell survival in glycerol while > 90% of the cells survived in DMSO and DEG. At 10% concentration in glycerol 89.1 ± 3.0% cells survived while in DMSO and DEG only 56.0 ± 2.6% and 60.7 ± 3.5% cells survived, respectively. At 12% and 15% concentration, after the incubation period the glycerol treatment had 70.0 ± 2.6% and 9.0 ± 2.6% cells survival, respectively. For the subsequent cryopreservation trials detailed later, glycerol (final concentrations of 5%, 8%, 10% and 12%), DMSO and DEG (both with a final concentration of 5%, 8% and 10%) were selected (Table S3).

For *A. pacificum*, DMSO, EG and glycerol had the least impact on cell health (Table S4). At 5% CPA concentration, > 90% of cells survived in both DMSO and EG while glycerol had 83.0 ± 2.1% survival. Increasing the CPA concentration to 8%, DMSO had 74.3 ± 3.1% of the cells surviving while EG had 60.0 ± 3.0% and glycerol recorded 50.8 ± 1.0%, respectively. At 10% concentration, DMSO treatment had 25.2 ± 1.5% cells survival while glycerol had 19.6 ± 2.1% and no cells survived in EG. There was no cell survival in the selected CPAs at both 12% and 15% concentration. For the subsequent cryopreservation trials shown later, DMSO, EG and glycerol (all final concentrations of 5% and 8%) were selected (Table S4).

Lastly, for *Breviolum* sp. DMSO, EG and PVP were selected for these experiments (Table S5). At 5% CPA concentration, these CPAs had > 90% of cells surviving. When increasing the CPA concentration to 8% 83.0 ± 3.0% of the cells survived in DMSO while in PVP 97.0 ± 1.0% survived and EG had the lowest cell survival at 11.3 ± 0%. At 10% concentration, DMSO treatment had 13.0 ± 2.0% of the cell surviving while PVP had 81.0 ± 2.0% and no cells survived in the EG treatment. At 12% concentration, only the PVP treatment had cells surviving (21.7 ± 1.2%). There was no cell survival in any CPAs at 15% concentration. For the subsequent cryopreservation trials detailed later, PVP (final concentrations of 5%, 8% and 10%) followed by EG and DMSO (final concentration 5% and 8%, respectively) were selected (Table S5).

### Effects of cryoprotectant treatment on cell health—combined CPAs

**Figure 1:**
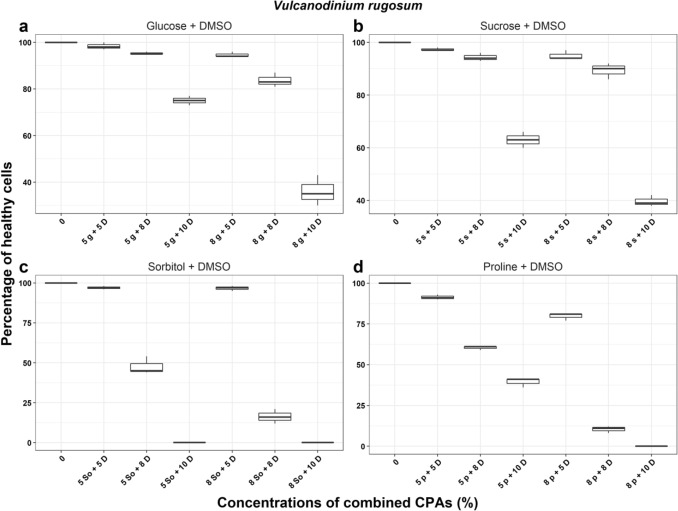
The percentage of healthy *Vulcanodinium rugosum* cells after treatment with combined cryoprotectant agents (CPAs) after one week incubation. *g* Glucose, *s* Sucrose, *D* Dimethyl sulfoxide, *So* Sorbitol, *p* Proline.

**Figure 2:**
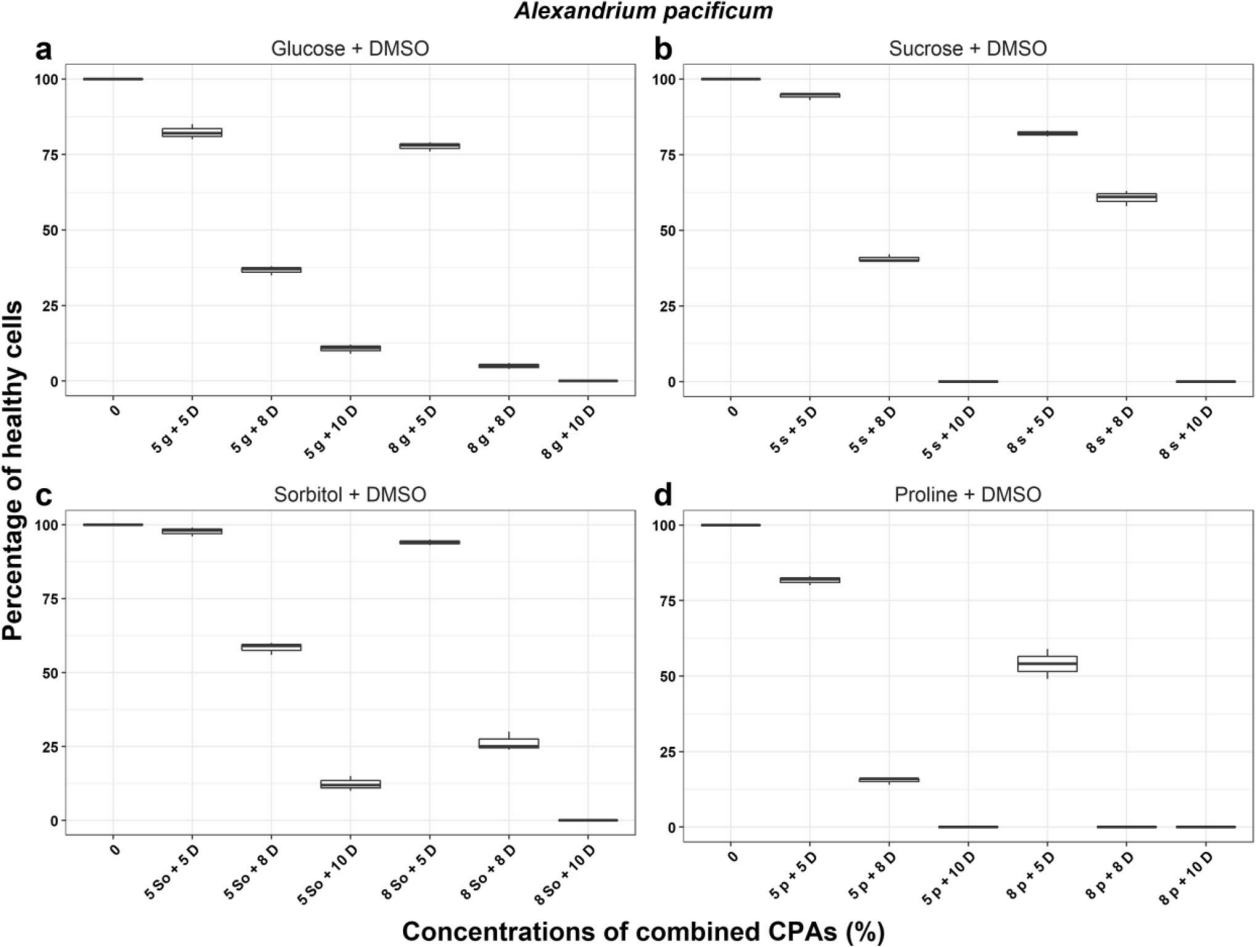
The percentage of healthy *Alexandrium pacificum* cells after treatment with combined cryoprotectant agents (CPAs) after one week incubation. *g* Glucose, *s* Sucrose, *D* Dimethyl sulfoxide, *So* Sorbitol, *p* Proline.

**Figure 3:**
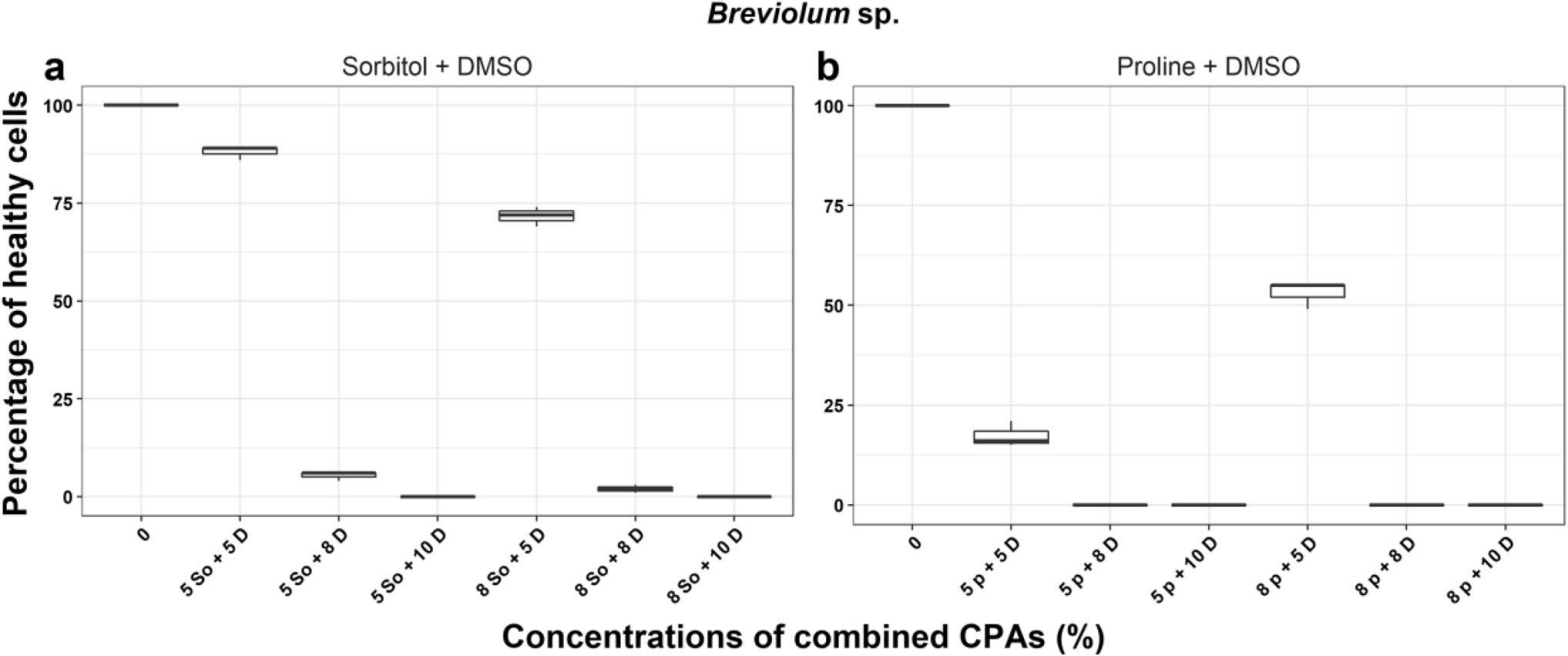
The percentage of healthy *Breviolum* sp. cells after treatment with combined cryoprotectant agents (CPAs) after one week incubation. *D* Dimethyl sulfoxide, *So* Sorbitol, *p* Proline.

In *V. rugosum*, treatments with sucrose + DMSO and glucose + DMSO resulted in between 36 and 99% of cells surviving after the incubation period” (Fig. 1). From these results, we selected 8% glucose, sucrose, and sorbitol + 5% DMSO for the cryopreservation trials described later. For *A. pacificum* the highest percentage of healthy cells surviving after the incubation period was 94.4 ± 1.5% recorded in 5% sucrose + 5% DMSO treatment. The best combination of CPAs was 5% sorbitol/sucrose + 5% DMSO and 8% sorbitol + 5% DMSO (Fig. 2) and these were selected for subsequent cryopreservation trials. Lastly, for *Breviolum* sp. the highest percentages of healthy cells surviving post treatments were in 5% sorbitol + 5% DMSO and 8% sorbitol + 5% DMSO with 88.0 ± 1.7% and 71.7 ± 2.5% cell survival, respectively (Fig. 3). 5% sorbitol + 5% DMSO and 8% sorbitol + 5% DMSO were selected as the preferred combined CPAs for subsequent cryopreservation trials. The full results of all other cryoprotectant treatments are shown in Supplementary Information Tables S6 to S8.

### Changes in cell size after cryoprotectant treatment tests

**Figure 4:**
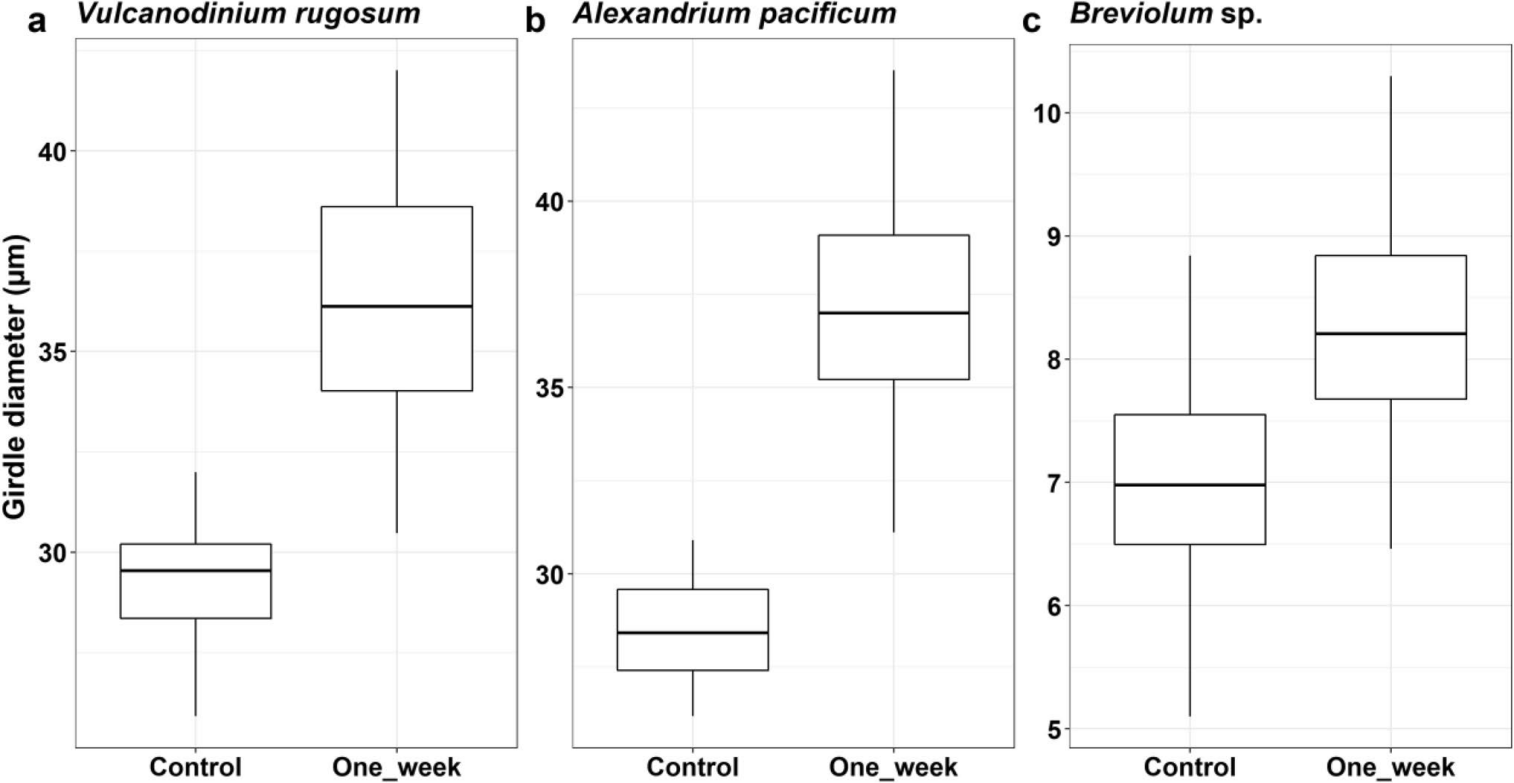
Changes in girdle size of each dinoflagellate species after one week of incubation with growth media without cryoprotectant agents (CPAs) compared to untreated cells (control). Results are shown for the preferred CPAs treatment for each species (n = 32–50).

Changes in girdle diameter were investigated during the recovery phase at one and two weeks for the three preferred CPAs for each species. At the end of the first week of recovery, the average girdle diameter of CPA treated *V. rugosum* cells significantly increased compared to untreated cells (control) (unpaired t-test, p < 0.05; treated cells: 36.3 ± 3.1 µm, untreated cells: 29.2 ± 1.5 µm; Fig. 4a). The same pattern was observed for CPA treated cells of *A. pacificum* during the recovery period. CPA treated cells were significantly larger than untreated cells (unpaired t-test, p < 0.05; treated cells: 37.2 ± 2.7 µm, untreated cells: 28.5 ± 1.2 µm; Fig. 4b). CPAs treated *Breviolum* sp. cells were also significantly larger than untreated cells during the recovery period (unpaired t-test, < 0.05; treated cells: 8.3 ± 0.9 µm, untreated cells: 7.0 ± 0.8 µm; Fig. 4c). At the end of week two, there were no significant differences (p < 0.05) between the median cell girdle diameters of the untreated (control) and the fully recovered treated cultures (*V. rugosum*: 29.4 ± 1.3 µm, *A. pacificum*: 28.7 ± 0.9 µm and *Breviolum* sp.: 6.8 ± 0.5 µm).

### Cryopreservation

While the combined CPA treatments showed considerable promise in initial experiments (Figs. 1, 2, 3), none of these combinations resulted in successful cryopreservation for any of the test species using either of the freezing techniques. Successful cryopreservation was only obtained by treatment with a single CPA.

#### Rapid freezing technique

*Breviolum* sp. was successfully cryopreserved using this technique. The cell viability after thawing was 10.0 ± 2.8% when 15% DMSO was used as the CPA, and the culture took three weeks for cells to recover and start dividing. When 15% EG was used, the cell viability was 2.0 ± 0.7% (Table 2). After thawing, no viable cells were observed for *V. rugosum*, and *A. pacificum* from any of the CPAs selected (Table 2). For the control species *A. carterae,* the cell viabilities were 82.7 ± 8.3% and 89.7 ± 2.5% in both the 10% and 15% DMSO treatments respectively (Table 2).

**Table 2:** Cell viabilities after the rapid freezing and controlled-rate freezing techniques using the selected single and combined cryoprotectant agents (CPAs).

Species	Final concentrations of single and combined CPAs used for freezing	Methods used and cells viability (% ± SD) after freezing and thawing	
		Rapid freezing technique	Controlled-rate freezing technique
*Vulcanodinium rugosum*	DMSO and DEG: 5%, 8%, 10%, 12% & 15% Glycerol: 5%, 8%, 10%, 12% & 15% 8% glucose/sucrose/sorbitol + 5% DMSO	NVC	NVC
*Alexandrium pacificum*	DMSO, EG & glycerol: 5%, 8%, 10%, 12% & 15% 8% sorbitol + 5% DMSO and 5% sucrose/sorbitol + 5% DMSO	NVC	NVC
*Breviolum* sp.	DMSO: 5%, 8%, 10%, 12% & 15% EG: 5%, 8%,10%,12% & 15% PVP: 5%, 8%, 10%, 12% & 15% 5% sorbitol + 5% DMSO and 8% sorbitol + 5% DMSO	10.0 ± 2.8% (15% DMSO) 2.0 ± 0.7% (15% EG)	45.4 ± 2.2% (15% DMSO) 17.4 ± 1.2% (15% EG)
*Amphidinium carterae* (positive control)	DMSO: 10% & 15% as the best concentrations^5^	82.7 ± 8.3% (10% DMSO) 89.7 ± 2.5% (15% DMSO)	44.1 ± 2.1% (10% DMSO) 65.2 ± 12.3% (15% DMSO)

*NVC* No viable cells observed, Values are mean percentage of three replicates ± standard deviation (SD) of the mean. *DMSO* Dimethyl sulfoxide, *DEG* Diethylene glycol, *EG* Ethylene glycol.

#### Controlled-rate freezing technique

**Figure 5:**
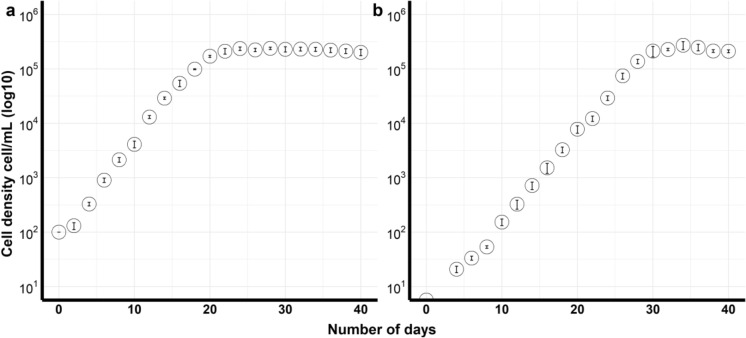
Growth curves of *Breviolum* sp. cultures with standard error bars. (**a**) Control (non-cryopreserved) culture, (**b**) Cryopreserved culture.

*Breviolum* sp. was successfully cryopreserved with the highest cell viability of 45.4 ± 2.2% in 15% DMSO. The culture took about six days to recover and start dividing (Fig. 5). When 15% EG was used the cell viability was lower, 17.4 ± 1.2% (Table 2). No viable *V. rugosum* and *A. pacificum* cells were observed after thawing in any of the CPAs selected in the experiment (Table 2). *Amphidinium carterae* cells were successfully cryopreserved with 10% and 15% DMSO treatments with cell viabilities of 44.1 ± 2.1% and 65.2 ± 12.3%, respectively (Table 2).

### *Breviolum* sp. growth curve post-thaw

For the non-cryopreserved *Breviolum* sp., the maximum growth rate was 0.71 divisions day^−1^ and the maximum cell density was 2.4 × 10^5^ cells mL^−1^ after 20 days in culture. The maximum growth rate for the cryopreserved cells was 0.65 divisions day^−1^ and maximum cell density 2.7 × 10^5^ cells mL^−1^ after 30 days (Fig. 5). There was no significant difference between the maximum growth rate or cell density between the cryopreserved and the non-cryopreserved cultures (Wilcoxon test, p > 0.05), however there was a delay of about six days in the growth of cryopreserved cells (Fig. 5).

## Discussion

The establishment of a common protocol for the cryopreservation of all microalgal species has proved very difficult^31^. In this study, a range of 12 different CPAs and two freezing techniques were tested to optimize a cryopreservation protocol for three morphologically and taxonomically diverse dinoflagellate species. Most dinoflagellate species that have large cell sizes and produce toxins have been difficult to cryopreserve^5^. For successful cryopreservation, adequate dehydration of the microalgal cells has to occur to prevent the formation of large intracellular ice crystals that kill cells during freezing^49^. To induce cell dehydration prior to freezing, a suitable type and concentration of a cryoprotectant for each microalgae species has to be selected^30^.

### Cryoprotectant agent assessments

In the first part of this study, a range of single penetrating and non-penetrating CPAs and different concentrations of combined CPAs commonly used in microalgal cryopreservation were tested^35^*.* Three most widely preferred single penetrating CPAs; DMSO, glycerol and methanol^30,35^ and five other common CPAs were assessed. In selecting the preferred CPAs for use in the cryopreservation experiments, we treated the three dinoflagellate strains with eight different single CPAs with concentrations varying between 5 and 15%, and different concentrations of combined CPAs. After the CPA treatment experiments, we observed that the CPA with the least impact on cell health varied between the dinoflagellate species. For example, EG was detrimental to cell health of *V. rugosum*, but it was one of the preferred CPAs for *A. pacificum* and *Breviolum* sp. These differences in percentage cell survival in different CPAs shows that the best CPA and its optimum concentration has to be determined empirically^35^. DMSO as a single CPA had no detrimental effects on cell health for the three test species and was preferred because it penetrates cell membranes easily, it can be removed easily from the cells and it did not cause bacterial contaminations during the incubation period^30,50^.

Our results regarding the preferred CPA varied from the findings of other studies. Santiago-Vázquez et al.^41^ and Li et al.^26^ showed that species from clade B, currently referred to as the genus *Breviolum*, could be cryopreserved using MeOH and PG but in our study, these two CPAs had negative effects on cell health. A possible reason for this is the one-week incubation period used during the CPA treatment might have been too long and the prolonged exposure in our procedure could have resulted in PG and MeOH accumulating in the intracellular space causing chemical toxicity affecting cell metabolism and health. The incubation of the PG and MeOH in treated *Breviolum* sp. cultures under minimal light (56 µmol m^−2^ s^−1^ PAR) may have initiated photosynthesis which allowed the CPA to induce potential toxicity thus damaging the chlorophyll pigments in the cells^30^. The *Breviolum* sp. cultures were non-axenic and treatment with PG also encouraged high bacterial contamination that might have negatively affected the health of the cells during the incubation period. However, the use of an antibiotic mix to reduce bacterial contamination in the PG treated *Breviolum* sp. cultures had no effect, suggesting bacterial contamination was not important here.

The combination of DMSO with non-penetrating CPAs caused very high bacterial contaminations in initial tests with all the treatments and the bacteria overgrowth might have limited the penetration of light into the cells and competed with the dinoflagellates for nutrients in the media. Certainly, high bacterial contaminations following CPA treatment can negatively influence the growth and recovery of cells^36^. During the incubation period with these CPAs, the dinoflagellates settled at the bottom of the well plates and dead microalgal and bacterial cells in the wells may have caused the conditions in the wells to be toxic affecting the cell health of our study species. Other CPAs that encouraged bacterial contaminations were glycerol, PEG, PG and PVP. To counter this, different concentrations and combinations of antibiotics were assessed, and a mixture of Penicillin-G, Gentamycin and Streptomycin was selected which effectively reduced bacterial overgrowth.

After the CPA treatments, the cultures were incubated with fresh media without CPA to support cell recovery. At the end of the first week of recovery, the mean girdle diameter of CPA treated cells was significantly greater than untreated cells. The percentage increase of girdle diameter in *V. rugosum* was 24.3%, 30.5% in *A. pacificum* and 18.6% in *Breviolum* sp. *Alexandrium pacificum* had the largest percentage increase in girdle cell diameter among the study species. The reason for this is not clear but we believe it is related to the morphological characteristics of this species that governs penetration and elimination of the CPA. Increased levels of CPA within the cells would increase the osmolarity of the cytoplasm causing the cells to take in more water from the growth medium by osmosis^51^. But at the end of this first week of recovery (when the girdle diameter was measured), most of the CPA should have been eliminated from the cells, and its osmotic effect would be limited. Perhaps the increase in cell size is due to cell growth without cell division during this phase. This idea is supported by the observation that after a second week of recovery, the cells had divided, and healthy cultures had been established with cells returning to their normal size. In addition, cryopreserved *Breviolum* sp. cultures took about six days before cell numbers started to increase, further suggesting that mitosis is inhibited until the CPA is eliminated from the cells^52,53^.

### Cryopreservation experiments

During freezing experiments, the CPA was added gradually in 100 µL aliquots to the culture before cryopreservation and removed after thawing to minimize osmotic stress that might affect the cell organelles and their membranes especially when using penetrating cryoprotectants like DMSO^5,35^. From the single CPAs selected, 15% DMSO was the best for cryopreserving *Breviolum* sp., and the culture was able to recover more easily and grow normally with no bacterial contamination after thawing. When 15% EG was used, the cell viability of *Breviolum* sp. was low after thawing and this could be due to their different cryoprotective efficiency in this species with DMSO being the more favourable CPA^35^.

None of the single CPAs were successful in cryopreserving *V. rugosum* or *A. pacificum* cells. Some dinoflagellate species have a thick thecal covering around their cells and the surface can be smooth or sculptured^46^. *Vulcanodinium rugosum* and *A. pacificum*, are both covered by heavy thecal plates. The presence of a rigid and impermeable cell wall in some microalgae may prevent the CPAs from penetrating into the cells^34^, and this may result in the formation of large ice crystals inside the cells during freezing. The cell sizes of *V. rugosum* and *A. pacificum* are relatively large, which could have prevented the CPAs from equilibrating the intracellular space before freezing. *Breviolum* sp. and *A. carterae* have smaller cell sizes, which may allow sufficient equilibration of CPAs to enable them to survive freezing. Smaller species of dinoflagellates survive cryopreservation more successfully than larger species from the same genus^5^, which suggests that the cell size of the microalgae can affect its success rate during cryopreservation.

Combined CPAs have resulted in higher survival rates during cryopreservation of the brown alga *Ectocarpus*^54^, the microalgae *Chlorella vulgaris*, *Nannochloropsis oculata* and *Tetraselmis tetrathele*^49^. However, the combined CPAs that were used in this study did not yield any successful results in any of the dinoflagellates. This might have been because the 30 min pre-incubation period we used did not allow sufficient time for the combined CPAs to reduce the size of ice crystals formed during freezing.

The optimum exposure time to CPA prior to freezing varies among species^30^. Sometimes a short exposure is ineffective and sometimes a lengthy exposure to CPAs may lead to toxicity in the cells^30^. Alternatively, when the high salinity media commonly used for culturing marine dinoflagellates are combined with non-penetrating (high osmolarity) CPAs, the resulting osmotic shock may affect many cellular functions and result in cell death. During cryopreservation, the concentration of the preferred cryoprotectant and its toxicity differs among microalgae species. To determine the best CPA concentration for our species, we chose a 1-week exposure period as described in Tzovenis, et al.^52^ which provides sufficient time to assess the full potential toxicity of the different CPAs. After cryopreservation, the cultures were kept for four days in liquid nitrogen. After thawing, they were incubated with a small amount of media for three days before finally adding excess media to dilute the CPAs. This allowed us to establish if the cells could recover and remove the excess CPA.

This study explored two main freezing techniques: rapid freezing and the controlled-rate freezing (temperature controlled). For the rapid freezing technique, various concentrations of the preferred single and combined CPAs were used for all our experimental dinoflagellates. *Breviolum* sp. was cryopreserved using this technique but after thawing the culture took a longer period of about up to three weeks to recover and re-stablish. Tanniou, et al.^33^, suggested that the rapid decrease in temperature upon immersion into liquid nitrogen, results in the formation of intracellular ice in some microalgae cells causing cell damage due to strong mechanical stress during rapid freezing techniques. Temperature controlled freezing was the best method for cryopreserving *Breviolum* sp. with the highest post-thaw cell viability. Saadaoui et al.^55^ showed the slow cooling rate applied in the controlled rate freezers minimizes the formation of large damaging ice crystals and this increases the survival of some species during freezing. The differences in cell viabilities from the two freezing methods shows that freezing and thawing protocols vary from one species to another and it is unlikely that a common universal cryopreservation protocol for all microalgal species could be developed^31,41^. Whole transcriptome analysis may reveal differential expression of certain functional genes^56^ that may be affected by the cryopreservation procedures (for example genes involved in the synthesis of fatty acids). The analysis of these genes in the species that failed to cryopreserve (*V. rugosum* and *A. pacificum*), compared to *Breviolum* sp. which did cryopreserve, will enable us to devise better approaches to their cryopreservation in the future. However, this approach was beyond the scope of the present study.

The ultimate goal of any cryopreservation experiment is to allow for the microalgal culture to retain its reproductive ability after thawing^57^. After thawing, fresh growth media was gradually added in small quantities to minimize osmotic shock in the cells as the media penetrated into the cells and replaced the CPA. The cultures were then incubated overnight in the dark at room temperatures to avoid possible light-induced damage by CPAs like DMSO^30^. A further incubation under red light for 48 h allowed enough time for the cells to eliminate the CPA before being transferred to normal growing conditions. The viabilities of cryopreserved *Breviolum* sp. cultures were assessed after the fourth day in their normal growing conditions rather than directly after thawing, to avoid overestimating cell survival^30,52,58^.

After thawing, the low number of cells prevented the application of Pulse Amplitude Modulation (PAM) fluorometry for assessing cell heath. After cryopreservation of *Breviolum* sp., it was not feasible to use flow cytometry to assess the health of the cultures, as the presence of bacteria and cell debris from broken cells after thawing affected the accuracy of the flow cytometer reading. Similarly, the presence of bacteria in the cultures meant that a cell viability bioassay^59^ was not viable. Other indirect methods of estimating viability such as cell motility and oxygen evolution may significantly overestimate the recovery potential of a species^30^. Gwo et al.^57^, states the most accurate method for determining the success of a cryopreservation experiment is to enumerate the cells using microscopy and compare this to a non-cryopreserved culture. Therefore, we assessed the reproductive ability of our species through cell counts every two days. For microalgae, allowing cells to actively divide and regrow back into a healthy culture is the most accurate measure of viability and should be used where feasible after cryopreservation experiments^30^. The microalgal cells should remain viable and grow normally into a healthy culture in the same way as non-cryopreserved cultures. In *Breviolum* sp., the culture experienced a post-thaw exponential growth phase from day six up to day 30 after freezing using the controlled-rate freezer, before attaining stationary phase. The growth pattern was equivalent to a culture that had not been cryopreserved with no significant differences in their maximum growth rates.

## Conclusion

*Breviolum* sp. has been threatened by increased global warming and high sea surface temperatures, which may affect its endosymbiotic relationship with the host corals^60^. These challenges in their natural habitats may cause *Breviolum* sp. cells to lose their genetic integrity, experience high cell mortalities or even extinction. Establishing a long-term preservation method for this species may assist in future research to mitigate or further understand these risks*. Breviolum* sp. was successfully cryopreserved using DMSO and after thawing, the cells were able to grow and divide into healthy a culture. The optimized cryopreservation protocol described in this study may also enhance the successful cryopreservation of other species in the genus *Breviolum* and members from other clades in the family Symbiodiniaceae. This will protect their genetic integrity for future research aiming to facilitate coral restoration efforts following coral bleaching and habitat destruction.

## Supplementary Information

Supplementary Information.

## Data Availability

All data generated or analysed during this study are included in this published article and its Supplementary Information files.
