# Supplementary material for: Cryoprotectant treatment tests on three morphologically diverse marine dinoflagellates and the cryopreservation of Breviolum sp. (Symbiodiniaceae)
Source: Sci Rep. 2022 Jan 13;12:646. doi: 10.1038/s41598-021-04227-2 (PMC8758677; doi:10.1038/s41598-021-04227-2)
Supplement: Supplementary file 1 — Supplementary Information. [file 41598_2021_4227_MOESM1_ESM.pdf]

Supplementary Information

Cryoprotectant treatment tests on three morphologically diverse marine dinoflagellates and the cryopreservation of *Breviolum* sp. (Symbiodiniaceae)

Joseph Kanyi Kihika*[,1,2], Susanna A. Wood[2], Lesley Rhodes[2], Kirsty F. Smith[2,3], Lucy Thompson[2], Sarah Challenger[2] and Ken G. Ryan [1]

[1] School of Biological Sciences, Victoria University of Wellington, PO Box 600, Wellington 6140, New Zealand
[2] Cawthron Institute, Private Bag 2, Nelson 7042, New Zealand
[3] School of Biological Sciences, University of Auckland, Private Bag 92019, Auckland 1142, New Zealand

* Correspondence: joseph.kihika@.vuw.ac.nz

Table S1. Antibiotics tests on non-axenic *Vulcanodinium rugosum*, *Alexandrium pacificum* and *Breviolum* sp. strains treated with cryoprotective agents (CPAs) that are associated with excessive bacterial growth.

**Antibiotic type**	**Concentrations used (10^{-6} g 10^{-3} L)**				
Ampicillin	50	75	100	125	150
Penicillin-G	50	75	100	125	150
Streptomycin	50	75	100	125	150
Gentamycin	25	50	75	100	125
Ciprofloxacin	5	7.5	10	12.5	15
Ampicillin + Streptomycin	50 +50	75 + 75	100 + 100	125 + 125	150 + 150
Penicillin-G + Streptomycin	50 +50	75 + 75	100 + 100	125 + 125	150 + 150

Table S2. Bacterial overgrowth in *Vulcanodinium rugosum, Alexandrium pacificum* and *Breviolum* sp. cultures after cryoprotective agents (CPAs) treatment tests. DMSO = Dimethyl Sulfoxide, PVP = Polyvinylpyrrolidone, PEG = Polyethylene glycol, EG = Ethylene glycol, PG = Propylene glycol, MeOH = Methanol, DEG = Diethylene glycol. **Legend: Yes** = cloudy cultures, **No** = clear cultures.

**Cryoprotective agents test**		**Presence or absence of bacterial overgrowth**
**1**	Glycerol treated culture	Yes
	Proline + DMSO, Sucrose + DMSO, Glucose + DMSO, Sorbitol + DMSO, PEG, PG and PVP treated cultures	Yes
**2**	Cell cultures from (1 above), treated with (Ampicillin, penicillin-G, gentamycin, ciprofloxacin, and streptomycin separately)	Yes
**3**	CPAs from 1 above, treated cultures **with** antibiotic mix	No
**4**	DMSO, MeOH, DEG and EG treated cultures **without** antibiotic mix	No
**5**	Control cultures (without CPA, without antibiotics)	No

Table S3. Percentages of healthy *Vulcanodinium rugosum* cells after treating with different single cryoprotective agents (CPAs). DMSO = Dimethyl Sulfoxide, PVP = Polyvinylpyrrolidone, PEG = Polyethylene glycol, EG = Ethylene glycol, PG = Propylene glycol, MeOH = Methanol, DEG = Diethylene glycol. **Values** are mean percentage of three replicates ± standard deviation (SD) of the mean.

**Cryoprotective agents (CPAs)**	**Percentages of healthy cells at different final CPAs concentrations used.**				
	**5%**	**8%**	**10%**	**12%**	**15%**
DMSO	100	97.3 ±1.5%	56.0 ±2.6%	0	0
MeOH	93.6±4.0%	7.0±3.6%	0	0	0
Glycerol	100	99.7±0.6%	89.1±3.0%	70.0±2.6%	9.0±2.6%
PG	99.3 ±0.0%	61.0 ±3.0%	0	0	0
DEG	100	91.0 ±2.6%	60.7 ±3.5%	0	0
EG	1.3±0.6%	0.7±0.6%	0	0	0
PVP	99.3±0.6%	46.3±5.5%	0	0	0
PEG	80.6±2.1%	1.0±0.0%	0	0	0

Table S4. Percentages of healthy *Alexandrium pacificum* cells after treating with different single cryoprotective agents (CPAs). DMSO = Dimethyl Sulfoxide, PVP = Polyvinylpyrrolidone, PEG = Polyethylene glycol, EG = Ethylene glycol, PG = Propylene glycol, MeOH = Methanol, DEG = Diethylene glycol. **Values** are mean percentage of three replicates ± standard deviation (SD) of the mean.

**CPAs**	**Percentages of healthy cells at different final CPAs concentrations.**				
	**5%**	**8%**	**10%**	**12%**	**15%**
DMSO	96.0±0.6%	74.3±3.1%	25.2±1.5%	0	0
MeOH	25.3±1.5%	0	0	0	0
Glycerol	83.0±2.1%	50.8±1.0%	19.6±2.1%	0	0
PG	39.9±1.2%	32.1±2.5%	0	0	0
DEG	19.7±2.0%	10.9±1.0%	5.0±1.0%	0	0
EG	95.7±1.0%	60.0±3.0%	0	0	0
PVP	79.7±1.5%	20.8±1.0%	6.3±2.1%	0	0
PEG	79.0±2.6%	9.0±2.6%	0	0	0

Table S5. Percentages of healthy *Breviolum* sp. cells after treating with different single cryoprotective agents (CPAs). DMSO = Dimethyl Sulfoxide, PVP = Polyvinylpyrrolidone, PEG = Polyethylene glycol, EG = Ethylene glycol, PG = Propylene glycol, MeOH = Methanol, DEG = Diethylene glycol. **Values** are mean percentage of three replicates ± standard deviation (SD) of the mean.

**CPAs**	**Percentages of healthy cells at different final CPAs concentrations used.**				
	**5%**	**8%**	**10%**	**12%**	**15%**
DMSO	98.3±1.2%	83.0±3.0%	13.0±2.0%	0	0
MeOH	0	0	0	0	0
Glycerol	11.3±2.1%	0	0	0	0
DEG	92.3±1.5%	3.0±1.0%	0	0	0
EG	94.7±1.5%	11.3±0.0%	0	0	0
PG	22.7±1.5%	0	0	0	0
PVP	99.0±1.0%	97.0±1.0%	81.0±2.0%	21.7±1.2%	0
PEG	87.7±1.5%	8.0±1.0%	0	0	0

Effects of combined cryoprotective agents (CPAs) treatment on dinoflagellate strains.

Table S6. Proportion of healthy *Vulcanodinium rugosum* cells after treating with different combined cryoprotective agents (CPAs). DMSO = Dimethyl Sulfoxide. **Values** are mean percentage of three replicates ± standard deviation (SD) of the mean.

**Types of combined CPAs**	**Concentrations of combined CPAs**	**Percentage of healthy cells**
Proline + DMSO	5% Proline + 5% DMSO	91.3±1.5%
	5% Proline + 8% DMSO	60.3±1.2%
	5% Proline + 10% DMSO	39.3±2.9%
	8% Proline + 5% DMSO	79.7±2.3%
	8% Proline + 8% DMSO	10.3±1.5%
	8% Proline + 10% DMSO	0
Glucose + DMSO	5% Glucose + 5% DMSO	98.3±1.5%
	5% Glucose + 8% DMSO	95.3±0.6%
	5% Glucose + 10% DMSO	75.0±2.0%
	8% Glucose + 5% DMSO	94.7±1.2%
	8% Glucose + 8% DMSO	83.7±3.1%
	8% Glucose + 10% DMSO	36.0±6.6%
Sucrose + DMSO	5% Sucrose + 5% DMSO	97.3±0.6%
	5% Sucrose + 8% DMSO	94.3±1.5%
	5% Sucrose + 10% DMSO	63.0±3.0%
	8% Sucrose + 5% DMSO	95.6±1.7%
	8% Sucrose + 8% DMSO	89.3±3.1%
	8% Sucrose + 10% DMSO	39.7±2.1%
Sorbitol + DMSO	5% Sorbitol + 5% DMSO	97.0±1.0%
	5% Sorbitol + 8% DMSO	47.7±5.5%
	5% Sorbitol + 10% DMSO	0
	8% Sorbitol + 5% DMSO	96.7±1.5%
	8% Sorbitol + 8% DMSO	16.3±4.5%
	8% Sorbitol + 10% DMSO	0

Table S7. Proportions of healthy *Alexandrium pacificum* cells after treating with different combined cryoprotective agents (CPAs). DMSO = Dimethyl Sulfoxide. **Values** are mean percentage of three replicates ± standard deviation (SD) of the mean.

**Types of combined CPAs**	**Concentrations of combined CPAs**	**Percentage of healthy cells**
Proline + DMSO	5% Proline + 5% DMSO	81.1±1.5%
	5% Proline + 8% DMSO	16.2±1.2%
	5% Proline + 10% DMSO	0
	8% Proline + 5% DMSO	54.0±5.0%
	8% Proline + 8% DMSO	0
	8% Proline + 10% DMSO	0
Glucose + DMSO	5% Glucose + 5% DMSO	84.0±2.5%
	5% Glucose + 8% DMSO	37.8±2.1%
	5% Glucose + 10% DMSO	11.0±1.0%
	8% Glucose + 5% DMSO	77.9±4.0%
	8% Glucose + 8% DMSO	5.3±1.5%
	8% Glucose + 10% DMSO	0
Sucrose + DMSO	5% Sucrose + 5% DMSO	94.4±1.5%
	5% Sucrose + 8% DMSO	42.0±2.6%
	5% Sucrose + 10% DMSO	0
	8% Sucrose + 5% DMSO	81.9±2.6%
	8% Sucrose + 8% DMSO	60.7±2.5%
	8% Sucrose + 10% DMSO	0
Sorbitol + DMSO	5% Sorbitol + 5% DMSO	97.3±1.5%
	5% Sorbitol + 8% DMSO	58.6±2.6%
	5% Sorbitol + 10% DMSO	14.0±4.4%
	8% Sorbitol + 5% DMSO	94.0±0.6%
	8% Sorbitol + 8% DMSO	27.5±3.0%
	8% Sorbitol + 10% DMSO	0

Table S8. Proportion of healthy *Breviolum* sp. cells after treating with different combined cryoprotective agents (CPAs). DMSO = Dimethyl Sulfoxide. **Values** are mean percentage of three replicates ± standard deviation (SD) of the mean.

**Types of combined CPAs**	**Concentrations of combined CPAs**	**Proportion (%) of healthy cells**
Proline + DMSO	5% Proline + 5% DMSO	17.0±3.2%
	5% Proline + 8% DMSO	0
	5% Proline + 10% DMSO	0
	8% Proline + 5% DMSO	52.6±3.5%
	8% Proline + 8% DMSO	0
	8% Proline + 10% DMSO	0
Sorbitol + DMSO	5% Sorbitol + 5% DMSO	88.0±1.7%
	5% Sorbitol + 8% DMSO	5.3±1.2%
	5% Sorbitol + 10% DMSO	0
	8% Sorbitol + 5% DMSO	71.7±2.5%
	8% Sorbitol + 8% DMSO	2.0±1.0%
	8% Sorbitol + 10% DMSO	0

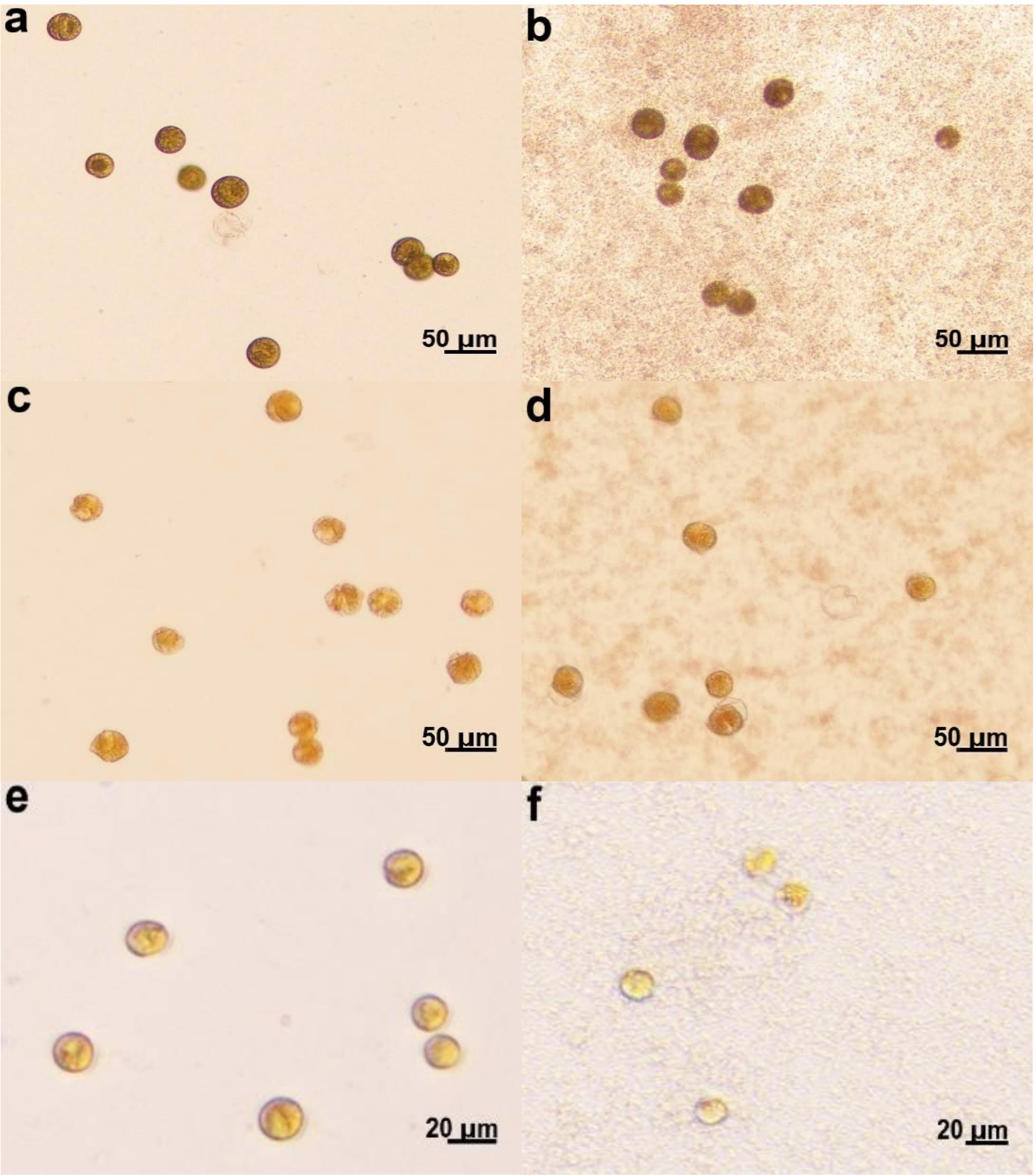

Figure S1. Reduced bacterial overgrowth after the addition of an antibiotics mix to the cryoprotective agents (CPA) treated dinoflagellate cultures: *Vulcanodinium rugosum* after treatment with cryoprotective agents (CPAs); (**a**), A clear culture with no bacterial contamination after addition of antibiotic mix (**b**), A cloudy culture due to bacterial contamination. *Alexandrium pacificum* when treated with CPAs; (**c**), A clear cell culture with no bacterial contamination after addition of antibiotic mix (**d**), A cloudy cell culture due to excessive bacterial overgrowth. *Breviolum* sp. cell culture when treated with CPAs; (**e**), A clear culture with no bacterial contamination after addition of antibiotic mix. (**f**), A cloudy culture due to excessive bacterial overgrowth (as in Table S2 above).

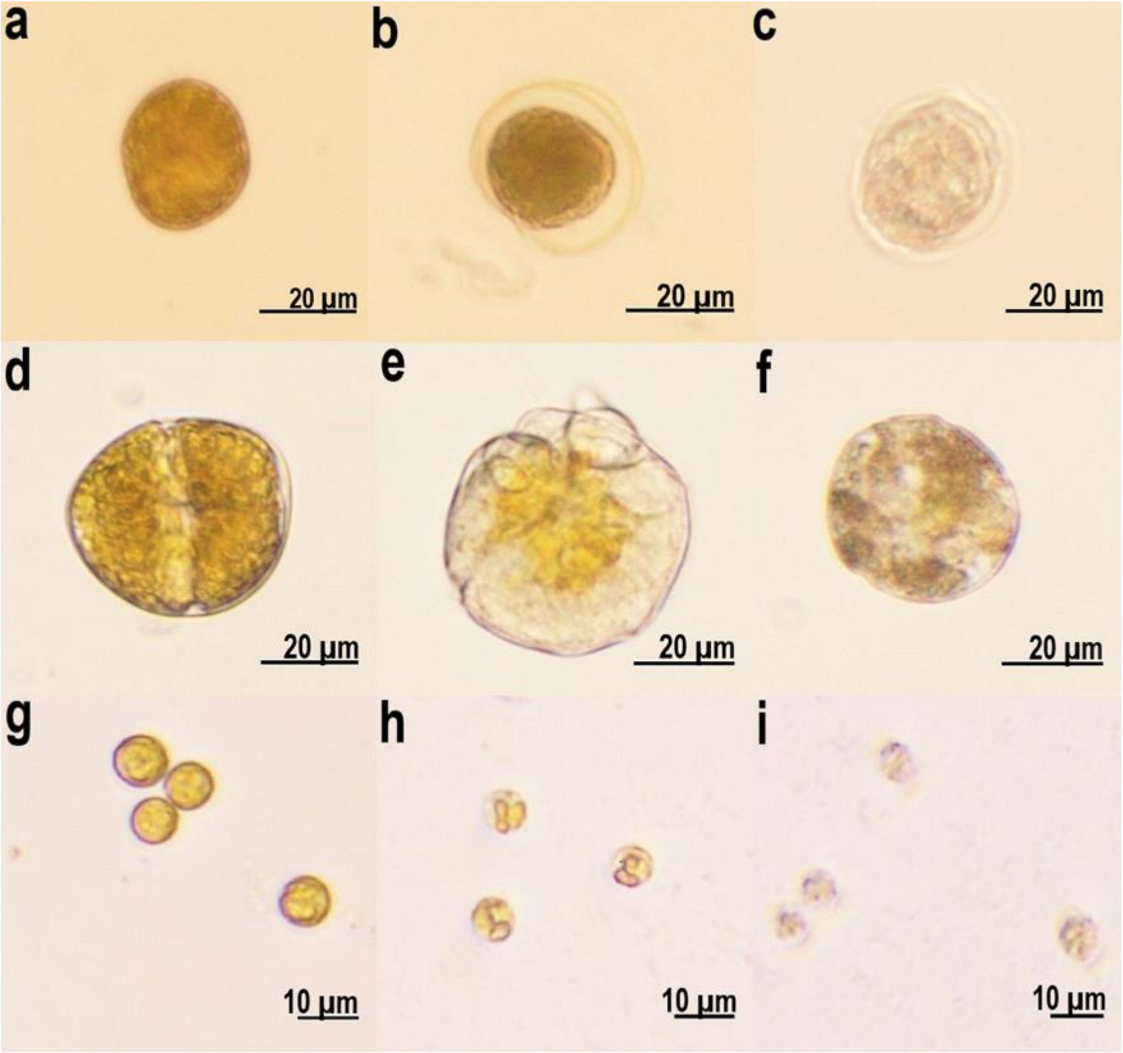

Figure S2. Typical cell morphologies of *Vulcanodinium rugosum* after one week incubation with cryoprotective agents (CPAs): (**a**)**,** healthy, (**b**), unhealthy, (**c**), dead. *Alexandrium pacificum* vegetative cells after one week incubation with CPAs. (**d**)**,** healthy, (**e**), unhealthy, (**f**), dead cell. *Breviolum* sp. vegetative cells after one week incubation with CPAs. (**g**)**,** healthy, (**h**), unhealthy, (**i**), dead.
